# Controlling control: toward a reflexive concept of officer safety

**DOI:** 10.3389/fsoc.2026.1644063

**Published:** 2026-03-03

**Authors:** Mario S. Staller, Swen Koerner, Benni Zaiser

**Affiliations:** 1 Department of Police, University for Police and Public Administration North Rhine-Westphalia, Cologne, Germany; 2 Department of Training Pedagogy and Martial Research, German Sports University Cologne, Cologne, Germany; 3 Independent Researcher, Aurora, ON, Canada

**Keywords:** control through insight, linear control, nonlinear thinking, Officer Safety 2.0, officer-induced jeopardy, police decision-making, reflexive policing, systems theory

## Abstract

Police responses to psychosocial crises frequently unfold in ambiguous, emotionally charged situations. While many encounters are handled professionally, others escalate, sometimes fatally. This paper observes such outcomes through a systemic lens rather than isolated individual decisions. Drawing on systems theory, we argue that the dominant model of officer safety, what we term Officer Safety 1.0, relies on three core assumptions: that safety is a producible condition, that violence is an external threat, and that thinking is guided by a linear, input–output logic concerning police-citizen interaction. These assumptions shape how officers perceive and act in complex situations, often reinforcing rigid and reactive forms of engagement that may inadvertently escalate risk. Officer Safety 1.0, in this sense, is grounded in a logic of linear control. We introduce an alternative model—Officer Safety 2.0—which retains the functional goal of control but reconfigures its foundation through reflexivity. In this model, safety is understood as a socially constructed fiction, violence as an interactive risk, and thinking is guided by a nonlinear understanding of social interactions. Within this model, control emerges from insight: the reflexive capacity to observe one’s own assumptions and their effects on the unfolding situation. Officers are encouraged not only to observe events, but also to reflect on the interpretive frameworks guiding their perceptions and actions. This reflexive approach aims to expand the behavioral repertoire available to officers, enabling more adaptive and situationally attuned responses that reduce the likelihood of escalation. Officer Safety 2.0 is not a replacement for existing tactics, but an educational framework that reframes them within a broader understanding of system logic and human interaction. It shifts the foundation of safety thinking from the pursuit of linear control to the cultivation of insight as a form of reflexive control.

## 1 Introduction

Police officers regularly engage with individuals in psychosocial crisis (PiC; Kyprianides and Bradford, 2024; Lorey and Fegert, 2021; Wittmann, 2022). In many instances, these encounters are handled professionally and without incident (Garner et al., 2018; Langton and Durose, 2013; Stroshine and Brandl, 2020), forming part of the routine processing of calls for service. However, there are also recurring cases where interactions with PiC result in tragic outcomes. For example, in Germany, incidents like the death of Mohammed Idrissi (Koerner et al., 2025) on June 18, 2020, or Mouhamed Lamine Dramé on August 8, 2022 (WDR Lokalzeit, 2024) gained considerable public, media, and scholarly attention. Yet beyond such visible incidents, there are numerous other cases (known to the authors through court records and professional experience) that did not end fatally and thus remained largely outside public discourse. These incidents may differ in visibility, but they share a common structure: they unfold in complex, ambiguous, and emotionally charged situations where police decision-making can tip the balance between resolution and escalation.

A closer examination of such cases reveal recurring dynamics that align with what has been described in the literature as officer-induced jeopardy (Bennell et al., 2022; Cyr, 2016; Staller et al., 2025a): at its core, this concept refers to non-necessary police actions that create a situation which then can only be resolved through the use of force (Cyr, 2016). The causes of such dynamics are often described as “unsound decisions” (Noble and Alpert, 2015) or “unwise decisions or actions” (Lee, 2021) made by officers that subsequently trigger a cascade of events potentially ending with avoidable bodily harm or even death. The central idea is that officers generate or passively accept unjustified risks or threats, which could and should be avoided. The concept of officer-induced jeopardy, particularly in legal discourse, focuses on the question of culpability: “[C]ulpability is inherent in the concept of officer-created jeopardy” (Stoughton et al., 2020, p. 156).

As a concept, officer-induced jeopardy—at least insofar as it is used in legal contexts—relies on a specific observer perspective. At its core, it is about causal attribution: Was the officer’s action the proximate cause of the situation that made the use of lethal force necessary? Was it an unsound decision or not? These questions are highly functional within legal discourse and are therefore entirely legitimate from that perspective.

For the purposes of the present paper, however, they are not the central focus. Drawing on systems theory (Luhmann, 1990, 1996), we contend that people (and thus police officers) see what they can see. As closed (psychological) systems, people know what they know. Our aim is therefore not to assess individual fault (which is always a matter of perspective), but to enhance the reflexive potential of individual officers, allowing them to see other options. To this end, we focus on a key concept that is not only practically relevant, but also communicatively accessible and institutionally anchored within police organizations: *officer safety*. We propose that rethinking officer safety beyond its conventional tactical framing offers a meaningful way forward for preventing tragic outcomes.

We begin with a system-theoretical reflection on police logics and how they construct a system-specific reality that is functionally oriented toward control (Section 2). We then turn to the concept of officer safety, which, within the framework of police logic, serves as a justificatory structure through which *control* is constructed in interactional situations. We discuss three conventional assumptions underlying officer safety, which reflect a legacy understanding of what we term Officer Safety 1.0 (Section 3). From there, we move to introduce a reflexive alternative to officer safety and formulate three revised assumptions that give rise to a different mode of control (Section 4). We conclude (Section 5) by articulating the concept of Officer Safety 2.0 as a shift from linear control to insight, arguing that reflexivity is the key ingredient for expanding alternative courses of action options available to police officers.

## 2 Police logics and police reasoning (of control)

From a systems-theoretical perspective (Luhmann, 1996), social systems operate according to distinct forms of sense-making that emerge from system-internal communication (Luhmann, 1990, 1996). These forms are not arbitrary; they are structured by the system’s guiding distinction—its code—which shapes how meaning is produced and maintained within the system. As the system’s internal logic, this code prefigures how thinking, decision-making, and action unfold. In the case of policing, we refer to this specific form of meaning-processing as police logic (Staller and Koerner, 2025c). Within the police system, this logic generates a system-specific reality that is reflected in internally constructed forms of “knowledge” about the police, about people, and about the world (Staller et al., 2023b). This knowledge is not only internalized through formal education and training structures, but also continually reproduced through curricula, tactics, and everyday police practices (Sierra-Arévalo, 2024; Thornton et al., 2012). The question of what the case is (in other words, the question of reality), is answered according to the system’s internal logic (Staller and Koerner, 2025b): The police sees, what the police can see.

This emphasis on distinctions can be specified with Spencer-Brown’s (1979) calculus of forms: social observation begins by drawing a distinction and indicating one side of it. In policing, the distinction dangerous/not dangerous frequently functions as such a primary form. Once indicated, this distinction structures what becomes perceptible as ‘relevant’ and what remains background. Importantly, the distinction can be re-entered into the situation: treating an encounter as ‘dangerous’ invites displays of control (e.g., commands, tactical positioning, weapon readiness) that may themselves be interpreted by the other party as threatening, thereby increasing the likelihood of agitation, resistance, or escalation. In this way, the distinction does not merely describe the situation; it can participate in producing the very dynamics it anticipates.

The system-specific reality of any organizational system, such as the police, emerges through selections that are contingent (yet not arbitrary) but rather shaped by the system’s internal structure within a functionally differentiated society (Luhmann, 2012, 2013). According to social systems theory (Luhmann, 1996), these selections are guided by a system’s code, its guiding distinction, which defines how external irritations are internally processed (Körner and Staller, 2023).

In Luhmann’s terms, policing is not a function system with a self-contained binary code of its own. Rather, police organizations are structurally coupled to the legal system and routinely operationalize the legal distinction of lawful/unlawful as a dominant reference for decision-making. At the same time, frontline encounters, especially crisis and violence-prone situations, are often processed through a more immediate operational distinction: dangerous/not dangerous. Within this context the distinction legality/illegality serves as a kind of (yet not exclusive) “police code”. We treat it as a legally anchored reference that is complemented (and in fast-moving encounters frequently pre-structured) by danger assessments that function as situational selectors.

These distinctions form the basis of the system’s operational logic and determines what counts as relevant information. To support this process, the system relies on selectors: heuristics that help determine which aspects of a situation should be given priority (Sierra-Arévalo, 2024; Zaiser et al., 2023). Empirical work in police psychology likewise shows that heuristic danger assessment is not merely an individual shortcut but can be trained, standardized, and institutionally embedded as an occupational routine (Ibrahim and Kattenberg, 2024).

Typical selectors in policing may include questions such as:

- Is there a threat to public safety or order or not?
- Has an offence been committed or is everything within legal bounds?
- Does someone require protection from violence or is no such danger present?

If no threat is identified, no legal violation has occurred, and no one appears to require protection, then the system has little to process. The system remains idle.

Consider the following example: German police students returning from practical training regularly report a recurring operational heuristic when dealing with individuals carrying knives (Staller et al., 2025a). The logic goes: *“A knife must be met with a gun.”* This formula illustrates how meaning is not derived from the situation itself, but from its interpretation within the police system. A knife is not merely an object in the world—it becomes, within the system’s logic, a proxy for illegality and immediate threat. The presence of a knife in any given situation activates specific selectors (*Is there a threat? Has an offence been committed? Does anyone require protection?*) and prompts a rapid internal calculation that constructs a coherent reality: the knife is likely to be interpreted as a sign of imminent danger, the presence of danger legitimizes the use of force, and the firearm is regarded as the appropriate tool to manage such danger.

This response is not simply the result of individual decision-making but stems from an institutional logic that reduces complexity by providing immediate, actionable interpretations. In this sense, the logic of *“knife = gun”* can be understood as a fixed chain of meaning and action that progresses in a preconfigured manner, leaving little room for contextual nuance or relational dynamics. Because of the underlying police logic, alternative constructions of reality remain inaccessible. For instance, the possibility that a knife is not being used in a threatening manner, when a person holding it may be experiencing extreme helplessness and trying to self-induce a state of dissociation by cutting themselves (Schauer and Elbert, 2010) is rarely integrated into the operational interpretation. The dominant logic narrows perception and action to what is already coded as relevant: illegality and threat.

Other documented manifestations of this police logic related to officer safety include the use of force against non-compliant, only passively resistant subjects (e.g., Klukkert et al., 2008; Sierra-Arévalo, 2019). In each case, specific behaviors are not interpreted in their potential multiplicity but are immediately subsumed under pre-established categories of threat, disorder, or criminal intent. A person who hesitates to follow an order is seen as resisting authority and someone who raises their voice is perceived as preparing for confrontation posing a threat to officer safety.

These examples demonstrate how police logic functions as a form of reduction of complexity: a systemic reduction of interpretive ambiguity through preconfigured patterns of perception and response. In doing so situational cues are rapidly filtered through institutional selectors and translated into standardized courses of action. Context-sensitive alternatives are not excluded because they are inexistant, but because they remain invisible within the system’s operational code.

From a systems-theoretical perspective, the dynamics described above, while consequential, are nonetheless functional (from the system’s internal view) and to be expected (Luhmann, 2000). A dysfunctional interpretation can be observed, when other systems (e.g., science, social work or public health) with other codes flesh out their point of view, i.e., their reality, of a given incident.

For example, the different and structurally functional viewpoints of different systems can be observed in the case of Mouhamed Lamine Dramé in Germany (Jüngst and Palm, 2024; Singelnstein et al., 2024; Staller et al., 2025a; Thompson, 2025). In addition to these scholarly accounts, further perspectives from different systems are documented in a journalistically investigative podcast produced by a public broadcasting station in Germany (WDR Lokalzeit, 2024), including an interview with the police officer who fired the shots (Voigt and Jaspard, 2024). In the interview, the officer stated that, within the situation, “I had no other choice.” This remark points to the fact that the officer’s actions were prefigured by the internal logic of policing, and that alternative perspectives may not have been available or conceivable in that moment.

However, as the diverse perspectives and the tragic outcomes of documented cases like Mouhamed Dramé in Germany suggest, there is an urgent need for reflection and change. If such outcomes are structurally expectable within the system, then the question arises: What is the function of the operational heuristics fueled by police logic currently propagated under the label of officer safety?

We argue that the core function of these heuristics is the production of control (Körner and Staller, 2023). In current practice, this control is largely configured as linear control: a mode of operation that follows a fixed and trivial input–output logic. In this context, any attempt to rethink police practice must ask: How can control be achieved without relying on the traditional, linear model of control? From a systems-theoretical perspective, this is a question about the functional equivalent of control. To approach this, we must first examine the assumptions that define how control is traditionally understood.

## 3 The form of control of Officer Safety 1.0

Our analysis begins with the observation that control constitutes a core functional form through which the police system reproduces itself (Körner and Staller, 2023). Within this context of control, the semantics of *officer safety* take on a particular significance. Officer safety not only expresses the police’s specific understanding of control. It also stabilizes and reinforces it. At its core, officer safety refers to the comprehensive self-protection of police officers. It is a recurring theme in learning objectives within police education and training and has become a focal point of scholarly reflection on policing (e.g., Blake et al., 2022; Buttle, 2007; Cunningham et al., 2024; Koerner and Staller, 2022b; White et al., 2025; Zaiser and Staller, 2015). As a semantic structure, officer safety is firmly anchored within the police system (Körner and Staller, 2023). Internal directives underscore its status as a guiding concept (e.g., for Germany: Leitfaden 371, 2025, e.g., for the United States: U.S. Department of Justice, 2022) and to this day, officer safety continues to generate a wide range of institutional discourse and practical anchoring (e.g., Avery, 2024; Füllgrabe, 2023; Herrnkind, 2022).

On an operational level, officer safety is typically framed as the tactically appropriate behavior to prevent or reduce threats to police officers during deployments (cp. Leitfaden 371, 2025; U.S. Department of Justice, 2022). On a more abstract level, however, the form of control becomes visible as the underlying logic: what counts as tactically appropriate, and which actions are deemed suitable to reduce danger depend on the prevailing understanding of control. And because control, as a form, does not determine its own content, its meaning can vary. The question of how situational control is achieved in the name of officer safety therefore depends on how control is conceptualized.

Yet control is not self-explanatory. The form of control can take on different content, depending on the assumptions that underlie it. The linear model of control, as identified earlier, provides the foundation for what we refer to as Officer Safety 1.0. To understand how Officer Safety 1.0 operates—and how it might be reconfigured without losing its core function—we must first examine the specific assumptions that currently define it. Put differently: if control determines the form of officer safety, then the assumptions shaping how control is understood and applied make up its content. In the following sections, we identify and examine three key assumptions that underpin the traditional model of officer safety:

1. Safety as a product.
2. Violence as an external danger.
3. Social interaction as a linear system.

### 3.1 Assumption 1: safety as a product

The first foundational assumption of Officer Safety 1.0 is that safety is a product, a condition that can be actively established through sound tactical behavior. Within this logic, safety is treated as a manageable variable in the operational environment, something officers can “create” through the correct combination of decision-making and action. In this view, the objective of Officer Safety 1.0 becomes the elimination of threat to restore a predefined condition of safety, typically articulated in spatial, procedural, or behavioral terms.

From a systems-theoretical perspective, however, safety is a “social fiction” (Luhmann, 1991, 2005). It functions as a semantic placeholder that creates the illusion of a stable referent while masking its (co-)constructed and contingent nature. It operates as a collectively shared expectation that reduces the complexity of future uncertainty. With regards to human agency, the concept of safety can be substituted by Luhmann’s proposition, that “there is no decision without risk” (Luhmann, 2005, p. 128). In this sense, the belief in safety as a controllable outcome conceals the fact that every decision to act (and not to act) involves a systemic exposure to risk, not the elimination of it.

Within policing, this fiction is rendered operational in several ways, like:

- heuristics (e.g., the 21-foot rule, which suggests that an individual with a knife or other edged weapon can cover 21 feet in the time it takes an officer to draw and fire their service pistol; Tueller, 1983),
- doctrines (e.g., the use-of-force continuum, which typically underlies law enforcement agencies’ use-of-force policies and outlines a continuum of escalating actions officers may take in response to the behavior they encounter, which, in turn, ranges from cooperative over resistant and assaultive to posing a threat to life or of serious bodily harm; National Institute of Justice, 2009), and
- tactical scripts (e.g., the 7 Tactical Principles, including cover, threat cues and Assessment, time-distance ratio, the plus one rule, verbalization and a winning, i.e., survival mindset; Walton, 2007).

These ways all present safety as something that can be engineered through appropriate responses. Such scripts reduce the concept of safety to a set of controllable inputs and outputs, thereby stabilizing the illusion of manageability. This belief in the producibility of safety results in a narrowed conception of agency: officers are trained to respond to what they perceive to disorder or danger. Yet the more fundamental question of how safety is defined, for whom, and under which systemic assumptions, remains largely unexamined within the framework of Officer Safety 1.0.

Moreover, the distinction between risk and danger—central to Luhmann’s theory of uncertainty (Luhmann, 1991, 2005)—remains largely absent from debates around Officer Safety 1.0. According to this distinction, danger refers to threats that originate from outside the system and are perceived as external to the acting subject, while risk refers to the possibility of harm resulting from one’s own decisions and actions. In contrast, the prevalent logic of the system tends to frame all uncertainty as external danger, requiring control and intervention. In doing so, it ignores the systemic risks that emerge from its own actions: risks generated internally by the threat or application of force, escalation strategies, or misread intentions. What remains unseen is that safety may be undermined not only by the world outside, but by the very logics through which the police seek to produce it.

This assumption is reflected in a wide range of police practices and training formats that present safety as the predictable outcome of correct behavior. These are not limited to dated concepts, such as the “10 deadly errors” (Brooks, 1976; Pokojewski, 1998) and other officer survival training content. Pre-attack indicators or perceptions of violent interpersonal cues as part of officer safety training (Johnson, 2016; Sweet et al., 2017), too, communicate the idea that fatal outcomes can result from individual mistakes and that safety, conversely, can be reliably produced through adherence to certain behavioral rules, such as maintaining distance, watching the hands, or never letting one’s guard down. Yet, such trainings fail to widen their threat-analytical lens to potential confounding factors of the perception of danger (Kahn et al., 2017).

A similar logic is evident in the deployment and justification of conducted energy weapons (CEWs). Marketed and trained as force avoidant (Axon, 2024), CEWs have repeatedly been shown to introduce, increase, and materialize potentially lethal risk (Vaughn et al., 2024; Williams et al., 2022). Furthermore, these devices suggest that safety can be technologically and, through it, reliably engineered, if “correctly” correctly (see, for instance, the use-of-force continuum; National Institute of Justice, 2009). This reinforces the belief that safety is a function of the right equipment applied under the right conditions. The assumption that the mere availability or application of a CEW can prevent harm reflects a broader confidence in the technological production of safety and the belief that complex human situations can be controlled with the push of a button, as if operating a machine.

### 3.2 Assumption 2: violence as an external danger

A second foundational assumption embedded in traditional officer safety is that the risk is “out there” (Koerner et al., 2023) and that it originates outside the police system (representing a danger; using the above-mentioned distinction between risk//danger). For example, concerning violence, risk is treated as an external feature of the environment: an act or intention located in the subject, the setting, or the situation itself. In this view, the role of the police is reactive: officers are there to contain, neutralize, or suppress violence that has already manifested or is about to emerge (Wall, 2020). This assumption has far-reaching implications for how officers perceive situations and interpret behavior. Violence becomes something to be anticipated in the other, for instance in the citizen who hesitates, the person in crisis who moves unpredictably, or the crowd that raises its voice. The other/s represent the danger (see, e.g., scholarly work on the police danger narrative; Eisenberg, 2023; Sierra-Arévalo, 2021; Sierra-Arévalo, 2024; Staller et al., 2023a; Staller and Koerner, 2022). This externalization of violence turns police attention outward, toward the identification of signs of danger, while minimizing attention to the relational or interactional character of the unfolding situation. Training scenarios often reinforce this framing. Officers are taught to scan for threat indicators, to maintain tactical positioning, and to act preemptively when certain cues appear, such as furtive movements, raised voices, or perceived non-compliance. These cues are read as evidence of impending aggression, and action is justified as a necessary response to protect oneself and others from external violence.

An example of this logic can be observed in how officers respond to individuals experiencing a mental health crisis. If a person is disoriented, unresponsive to commands, or displays erratic behavior, these actions are frequently interpreted as signs of volatility and danger (Ruiz, 1993; Wittmann et al., 2021). The behavior is read as a prelude to violence, rather than as a manifestation of distress, confusion, or altered perception. The situation is framed as one in which the officer must take control to protect themselves and others from a presumed external threat. The potential co-construction of escalation through misunderstanding, mismatched expectations, or lack of de-escalation training remains outside the officer’s frame of reference (Koerner et al., 2025). The result is a cognitive frame in which the self is shielded from scrutiny. The possibility that violence might emerge not solely from the subject, but also from the structure of the encounter or even from the officer’s own behavior remains largely invisible. In this logic, the officer does not participate in the creation of risk but merely reacts to a risk that is already “out there.”

It is worth noting, that this assumption is further stabilized by legal and procedural norms that frame use of force as a necessary consequence of non-compliance or resistance. Once violence is understood as external, the use of force is positioned as morally and institutionally justified, and the focus shifts to tactical effectiveness rather than interactional dynamics. Violence, in this framing, is something that happens to the police, not something that happens with them. It is a danger, not a risk.

### 3.3 Assumption 3: social interaction as a linear system

A third foundational assumption underlying Officer Safety 1.0 is the reliance on linear system thinking. This form of reasoning assumes that situations unfold in a predictable, sequential manner: a stimulus appears, it is assessed, and an appropriate response follows. The model is based on an input–output logic, in which the correct perception of a threat triggers the correct tactical behavior. Within this framework, cause and effect are tightly coupled, and complexity is managed through pre-scripted decision paths.

This way of thinking is operationalized in countless police procedures, which prescribe exact action steps that officers “shall” take in response to a precisely pre-defined situation (for an example, see the San Francisco Police Department’s 130 “General Orders” that govern officers’ daily conduct; San Francisco Police Department, 2025) and training models, including the use-of-force continuum, which considers an edged weapon as a lethal threat and, therefore, suggests lethal force by police (National Institute of Justice, 2009). Officers are taught to follow standardized response protocols, whether in traffic stops, building entries, or crisis interventions. The expectation is that, if the officer applies the appropriate procedure at the appropriate time, the situation will resolve in a controlled and desirable manner. Deviations from the expected sequence are interpreted as risk, or even as failure.

What this logic produces is a mode of engagement that filters out ambiguity. Situations must be rendered recognizable and assessable quickly; signals must be interpreted without delay. The emphasis on reaction speed, often reinforced through tactical training scenarios, means that slowness, hesitation, or reflective pause are treated as dangerous. Control, in this paradigm, is not negotiated but asserted through decisive and time-efficient action.

A typical manifestation of linear system thinking can be seen in response models that categorize situations into escalating levels of threat, each matched with a corresponding tactical response. Aside from the use-of-force continuum discussed above (National Institute of Justice, 2009), critical and major incident response plans exemplify this by laying out police response steps in a linear-binary nature. For instance, officers may be communicating with a PiC, who has locked themselves in their house, and be told that the PiC is unwilling to step out and be taken to a hospital for a psychiatric hold. Even if the PiC is de-escalatable and open to maintain communications, the refusal to step out or surrender their edged weapon may qualify this incident as a barricaded subject incident under the International Association of Chiefs of Police model procedure (IACP Law Enforcement Policy Center, 2020). This can trigger a full critical incident response that typically involves containment of the premise, along with the deployment of special weapons and tactics teams, which, in turn, might further intimidate the PiC and increase odds of a tactical rather than negotiated resolution, compared to affording initial communications more time to build trust and influence the PiCs behavior positively. In these models, behavioral cues are treated as triggers that move the situation from one level to the next and create a chain of events in which the officer’s role is to keep pace with the unfolding logic. The model leaves little room for relational feedback, circular dynamics, or second-order observation. The complexity of social interaction is flattened into a linear, path dependent avenue from stimulus to intervention. This assumption encourages officers to treat uncertainty not as a condition to be navigated but as a deviation to be corrected, ideally through quick and decisive control.

Taken together, these three assumptions constitute the core logic of Officer Safety 1.0. Safety is understood as a technical outcome that can be engineered, violence is seen as an external threat that must be contained, and processes follow linear, stimulus–response mechanisms. The result is a model of officer safety in which linear control becomes the preferred mechanism for managing uncertainty. When situations become unpredictable, emotionally charged, or ambiguous, officers operating within this logic are trained and expected to respond by reasserting order: quickly, decisively, and unidirectionally. In this paradigm, linear control is not merely one option among others; it becomes the dominant form through which safety is imagined, pursued, and justified. Understanding this logic is essential for any attempt to rethink officer safety beyond its current boundaries.

In the next section, we outline a reflexive alternative that seeks not to reject control, but to reconfigure its meaning and method in light of the systemic and interactional dynamics at play.

## 4 A reflexive policing approach to control

Our proposed alternative to the traditional account of officer safety is grounded in a reflexive approach to policing (Koerner and Staller, 2022c; Staller and Koerner, 2021, 2026). The core idea of this approach is that policing is never separated from the assumptions and conditions under which it operates and that these assumptions themselves shape how situations are perceived, interpreted, and acted upon. Reflexive policing incorporates second-order observation: not only are events and risks observed, but the act of observing—and the frameworks guiding that observation—are themselves subject to scrutiny.

This does not mean abandoning the assumptions of Officer Safety 1.0 but rather placing them under observation. Safety may still be understood as a product, but its constructedness becomes visible. Violence may still be perceived as external, but the implications of that framing are recognized. Situations may still be approached with linear models, but the limitations of such models are no longer invisible. In this sense, Officer Safety 2.0 retains the functional orientation of control, but seeks to revise and reconfigure its underlying logic. The model of control shifts from a linear mode to one grounded in insight: a form of control that emerges through reflexivity concerning ones observations. As such, the following sections proposes three alternative core assumptions to the assumptions of Officer Safety 1.0.

### 4.1 Reflexive assumption 1: safety as a fiction

In a reflexive policing framework, safety is no longer treated as a stable condition or a guaranteed outcome, but as a semantic structure that orients expectations under uncertainty. Rather than being something that simply *is* or *is not*, safety functions as a guiding fiction, a collectively held idea about what should happen, and what must be avoided. This fiction is not false; it is socially real, precisely because it shapes how decisions are made, how risks are weighed, and how situations are interpreted.

Luhmann (2005) referred to such concepts as semantic placeholders (“Leerbegriffe”): terms that have no empirical content of their own but operate by coordinating communication. In this sense, safety serves a valuable purpose: it makes it possible to act in situations that are inherently ambiguous. However, the reflexive turn involves making this logic visible. Officers are not simply oriented toward safety, but toward a particular idea of safety, one that is shaped by institutional semantics, professional cultures, and situational framing. A reflexive approach to officer safety encourages officers to ask not only “am I safe?,” but also “what does safety mean here?,” and “how is this idea of safety shaping what I see and do?” This move enables a shift in perspective: from treating safety as a fixed endpoint to approaching it as a temporarily stabilized expectation: one that can and must be re-examined in light of its practical consequences.

### 4.2 Reflexive assumption 2: violence as an interactive risk

Within a reflexive approach to officer safety, violence is not treated as a fixed attribute of a person or a scene, but as a risk that emerges in and through interaction. It is something that may or may not materialize, depending on how perceptions, communications, and actions unfold in relation to each other. Violence, in this view, is neither wholly external nor entirely controllable. It is contingent, relational, and sensitive to framing. What becomes critical, then, is not simply identifying whether violence is present, but understanding how the potential for violence is co-produced through gestures, language, tactical decisions, the use of space, and the mutual reading of intentions. From this perspective, officers are not simply responders to violence, but participants in situations where violence becomes more or less likely depending on how the encounter is structured.

This shift does not assign blame to officers, nor does it romanticize the dynamics of aggression. Rather, it opens space for recognizing the interactive nature of threat emergence, and for cultivating practices that take this dynamic into account. In this reflexive approach, violence is understood not only as something to be prevented, but as something whose conditions of possibility can be reflexively influenced, often subtly, through timing, tone, stance, or framing.

By observing how violence becomes possible—not just when or where—it becomes possible to identify leverage points for action that are not based on dominance or force, but on shaping the conditions under which force becomes unnecessary.

### 4.3 Reflexive assumption 3: social interaction as a nonlinear system

A reflexive approach to officer safety is based on the recognition that social interaction is not a linear system (Luhmann and Schorr, 1982; Staller et al., 2021). It is not governed by simple cause-and-effect relationships, but shaped through mutual adaptation, emergent dynamics, and recursive feedback. Actions taken by one actor influence the perception and behavior of others, often in unpredictable and compounding ways.

Linear models of decision-making that assume input leads to output in a clear and timely fashion offer the illusion of (linear) control. But they do so by reducing complexity: they filter out the inherent uncertainty and contingency of social interaction. In the context of pedagogy, Luhmann and Schorr (1982, 2015) referred to this problem as a “deficit of technology”: Unlike in technical domains such as computing, mechanics, or pharmacology, there is no reliable tool, app, or pill that produces predictable outcomes in social contexts. In policing, this technological deficit is often compensated for by introducing “finalized knowledge.” Finalized knowledge is condensed, simplified in both content and language, correct and useful, and stripped of uncertainty (Koerner and Staller, 2022a). In practice, this takes the form of causal heuristics or mechanistic scripts that promise control by identifying the “right” intervention at the “right” time. Yet these attempts to engineer certainty risk oversimplifying the relational complexity of the encounter.

Acknowledging the nonlinearity of social interaction also means recognizing that meaning and effect are context-dependent and temporally distributed. A gesture, a pause, or a shift in tone may alter the trajectory of an encounter, not because they cause a specific outcome but because they participate in shaping its emergent logic. This insight demands a shift in perception: away from linear scripts and toward context-sensitive forms of understanding and insight.

Reflexive policing, in this sense, is not about abandoning structure, but about understanding how structure interacts with contingency. A reflexive approach to officer safety does not only observe the observer but also apply control to the act of controlling and reconsiders how control is produced. Officers are thus invited to observe how their own actions enter the loop of meaning-making, not to predict every consequence but to remain open to the possibility that control may emerge through flexibility, timing, and relational attunement rather than through immediate closure (Staller et al., 2025b).

## 5 Thinking differently about officer safety: Officer Safety 2.0

In the previous sections, we outlined a reflexive alternative to the traditional model of officer safety. This alternative builds on the premise that the assumptions underlying police action, especially regarding safety, violence, and control, must themselves become the object of observation. We now give this alternative a name: Officer Safety 2.0.

**Figure 1:**
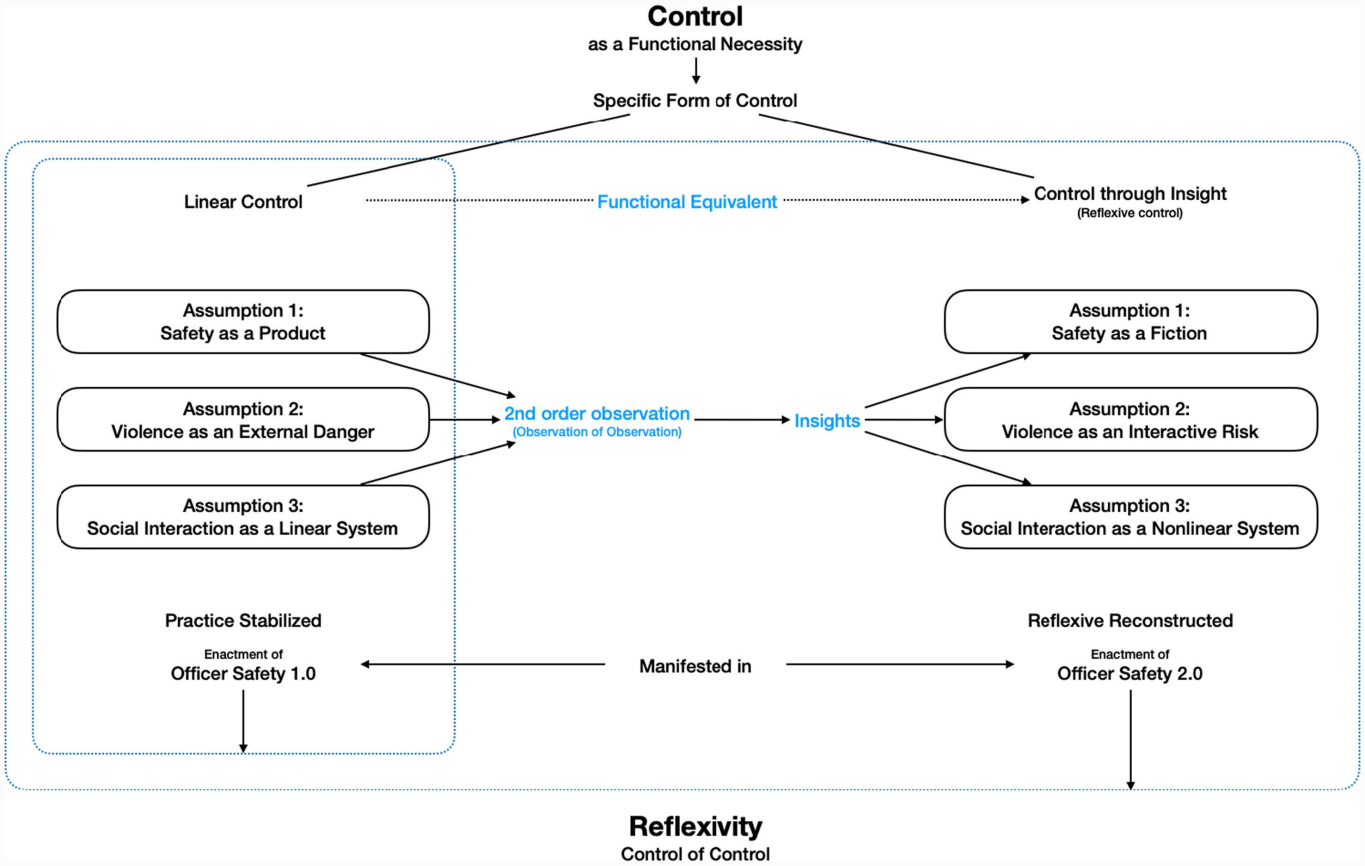
Officer Safety 2.0 as a reflexive reconfiguration of control.

Figure 1 visualizes the conceptual shift: Officer Safety 2.0 emerges through the reflexive transformation of Officer Safety 1.0. Officer Safety 1.0 is grounded in linear control, based on the assumptions that safety is a producible condition, violence is an external threat, and social interaction follows a linear logic. In contrast, Officer Safety 2.0 introduces a different operational logic: one that replaces linear control with insight. Insight, in this context, functions as a systemic equivalent to linear control. It allows officers to regulate their actions not by applying fixed procedures, but by recognizing and modulating how their own assumptions, perceptions, and actions shape unfolding interactions. It includes the ability to recognize how safety is constructed, how violence becomes possible, and how interaction unfolds in nonlinear and reciprocal ways. This form of control is not applied to a situation. It emerges within it.

The transformation from Officer Safety 1.0 to 2.0 proceeds through second-order observation: the capacity to observe how one observes. However, reflexivity in the full system-theoretical sense goes further. It refers to the control of control—to the ability of the system to re-enter its own operations and apply its own logic to itself. Officer Safety 2.0 operates at precisely this level: it enables policing to reflexively engage with its own logic of control.

Importantly, Officer Safety 2.0 does not replace Officer Safety 1.0. It contains it. The tactical and procedural knowledge of 1.0 remains vital but it is now situated within a broader architecture of reflexive understanding. While Officer Safety 1.0 seeks certainty and reduces ambiguity through linear routines, Officer Safety 2.0 accepts uncertainty as a structural condition and cultivates the competence to navigate it. It is not an abandonment of control, but a reconfiguration of what control can mean in complex human encounters.

As such, this model marks a shift from reactive execution to reflexive orientation. It introduces a new mode of control. However, Officer Safety 2.0 does not guarantee better outcomes. It provides a systemic potential: the possibility to observe and revise the very assumptions through which police officers stay safe. As such, Officer Safety 2.0 is not merely a tactical model. It is best understood as a concept of education (Staller and Koerner, 2025a) that moves beyond the training of techniques toward the cultivation of reflexive competence. It points to the need for professional development that goes beyond the transmission of rules and procedures. As such, Officer Safety 2.0 requires theory to make a practical difference.

Practically, Officer Safety 2.0 is not a new checklist but a set of reflexive micro-practices that can be integrated into routine operations without replacing tactical competencies. In everyday encounters, this includes (a) actively identifying the operative distinction currently guiding perception (e.g., dangerous/not dangerous; compliant/non-compliant), (b) briefly testing the distinction against alternative indications (What would count as evidence for the non-danger side?), and (c) considering how one’s own control displays enter the interactional loop and may shape the other party’s options. At the organizational level, Officer Safety 2.0 can build on existing second-order practices already present in many agencies, such as after-action reviews, supervisory debriefings, body-worn camera review, and crisis negotiation protocols, by making the underlying observation frames explicit rather than tacit.

However, potential limitations should be acknowledged, too: reflexive practices can increase cognitive load and may be misread as hesitation in time-critical situations; they also require supportive supervision and training designs to avoid becoming mere post-hoc rationalizations. Officer Safety 2.0 therefore complements rather than displaces Officer Safety 1.0: it preserves tactical readiness while adding an educational layer that expands the repertoire of situational control.
